# A New Synthesis for Dual Use Research of Concern

**DOI:** 10.1371/journal.pmed.1001813

**Published:** 2015-04-14

**Authors:** Michael J. Imperiale, Arturo Casadevall

**Affiliations:** 1 Department of Microbiology and Immunology, University of Michigan, Ann Arbor, Michigan, United States of America; 2 Department of Molecular Microbiology and Immunology, Johns Hopkins Bloomberg School of Public Health, Baltimore, Maryland, United States of America

## Abstract

Michael Imperiale and Arturo Casadevall propose a path forward for life sciences research whose results could be misused to cause harm.

**Summary Points**

- “Dual use research of concern,” or DURC, denotes beneficial life sciences research whose results could be misused by those wishing to cause harm.
- Dual use was largely ignored until a couple of years ago, when debate erupted over the publication of manuscripts describing enhanced transmissibility of H5N1 highly pathogenic avian influenza.
- Dual use has not served its intended purpose of clearly delineating research that might require additional scrutiny.
- Over the decade, the debate has largely switched focus from biosecurity to biosafety.
- We propose a path forward that we believe better defines the issues at hand.

In 2004, the National Research Council (NRC) published *Biotechnology Research in an Age of Terrorism* [1], which introduced the term "dual use dilemma" to denote beneficial life sciences research whose results could be misused to cause harm. That phrase evolved into “dual use research of concern” or DURC. The subsequent decade was characterized by a dichotomy in the response of scientists and the public, with both avid interest and complete disinterest. DURC was a largely specialized issue with awareness confined to a small group of experts in the scientific, government, and security communities until a vigorous and sometimes acrimonious debate erupted in 2011–2012 when two manuscripts reported the experimental derivation of mammalian transmissible H5N1 influenza [2,3]. Here, we examine a decade of dual use and propose a new synthesis for moving forward.

The modern era of concern about bioterrorism began in the 1990s, resulting in substantial funding in the biodefense area [4]. The reality of bioterror on United States soil began with the mailings of *Bacillus anthracis* spores to news outlets and elected officials after 9/11. The US government (USG) response to these attacks included designation of pathogens that were most likely to pose a threat in the Select Agents and Toxins List (SATL) [5] and the definition of, and commitment of significant funding to, National Institute of Allergy and Infectious Diseases priority agents.

Select agents were not the only pathogens drawing attention in the early 2000s. In 2001, a laboratory in Australia introduced the interleukin 4 (IL-4) gene into ectromelia virus in an effort to produce a contraceptive vaccine to control the wild mouse population. Surprisingly, the modified virus was more virulent than wild type in naïve animals and also virulent in immune mice [6]. Another study showed how poxviruses evaded the antiviral effects of the complement system, suggesting a means to engineer a hypervirulent variola virus [7]. In 2002, the poliovirus genome was assembled from oligonucleotides, raising concerns that a pathogen could be constructed from sequence information alone [8]. In 2005, a paper described modeling of vulnerabilities in the milk supply to contamination with botulinum toxin [9], and the 1918 influenza virus was reconstructed [10].

In 2005 the National Science Advisory Board for Biosecurity (NSABB) convened and produced reports dealing with various aspects of dual use research. A principle guiding the early deliberations was that a vibrant research enterprise is society’s best defense against misuse. It developed a framework for discussion, constructed a definition of dual use research of concern, and identified specific categories of experiments, based on a similar list in the NRC report, that were most likely to be worrisome [11]. Despite all this energy, there remained little enthusiasm for a broader discussion of dual use in the scientific community.

The greatest test of the NSABB framework began in fall 2011, when the Kawaoka and Fouchier laboratories submitted manuscripts reporting that H5N1 avian influenza virus could be made transmissible between mammals by aerosols [2,3]. The NSABB initially recommended publishing the general finding that H5N1 could acquire this phenotype while withholding details of the enabling mutations, which ignited a vigorous debate. The World Health Organization organized a conference, which was attended almost exclusively by influenza researchers, and recommended full publication [12]. Fouchier argued that the results had been misinterpreted [13]. The journals, *Science* and *Nature*, stated that they would only accept the NSABB recommendations if a mechanism were developed for sharing the details with public health officials and others with a need to know [14]. Some influenza researchers enacted a moratorium on similar experiments pending discussions about how best to proceed [15].

The USG convened an NSABB meeting in March 2012, at which members were informed that redaction would trigger export control regulations and that selective sharing was not feasible, effectively forcing a yes-or-no decision on whether to publish. The NSABB recommended that the Kawaoka and Fouchier manuscripts be published by unanimous and split votes, respectively. Concomitantly, the USG released a policy for dual use research oversight [16]. This policy covered 15 agents and toxins and seven categories of experiments, called for a risk-benefit assessment of the research, and required development of a risk mitigation plan. Missing was any indication of how risks and benefits should be quantified. In 2013, the National Institutes of Health issued a companion policy for institutional oversight of dual use research [17].

In the subsequent two years, the controversy simmered without any discussion or resolution of underlying scientific, ethical, biosafety, or biosecurity issues. After the moratorium ended in early 2013 [18], other manuscripts describing similar experiments with influenza virus appeared [19,20]. A paper describing a new strain of *Clostridium botulinum* producing a toxin not susceptible to the current antitoxin was published [21], and the journal decided not to include the toxin sequence, arguing that it would be irresponsible to do so until countermeasures were available [22]. Without clear governmental guidance, the responsibility to determine DURC has fallen on journal editors, usually resulting in publication.

In 2014 a vigorous new debate arose, fueled in part by revelations of incidents at the Centers for Disease Control and Prevention (CDC) involving *B*. *anthracis* and H5N1. This debate was carried out largely in the lay press, which published several articles critical of so-called “gain-of-function” (GOF) experiments. The debate became rapidly polarized: individuals concerned about biosafety issues formed the Cambridge Working Group, while scientists favoring freedom in scientific pursuit formed Scientists for Science. Despite the ferocity of the debate, the positions of both groups were remarkably similar, and some scientists signed on to both camps. Papers appeared arguing against GOF research in general, contending either that such experiments are unethical [23] or that the risk of accidental release of organisms is greater than that of misuse [24]. The latter point was not far-fetched since even in the highest containment labs such as the CDC, accidental exposures occur. In all, the discussion expanded from biosecurity concerns to wider-ranging issues, with a new focus on biosafety.

The situation today is highly unsatisfactory. The NSABB DURC definition was a major step forward, but it requires an assessment that is beyond the capabilities of editors and Institutional Biosafety Committees [25]. Experimental work that raises eyebrows is proceeding as scientists try to come to grips with the benefits and risks. There has been an absence of leadership by the USG, exemplified by the long slumber of the NSABB from 2012 to 2014 despite the issues raised by the H5N1 controversy and calls for dialogue from major societies such as the American Society for Microbiology.

A new element in the debate was the call for risk-benefit analysis prior to commencing this type of work. While we are both on record as supporting risk-benefit analysis [26], we caution that this approach alone is not a panacea. These analyses are difficult to perform because the values of the variables cannot be assigned with confidence. For example, it is difficult to gauge the value of basic research that may not have an obvious use for many years [27]. Similarly, risk assessments based on history may not reflect improvements in safety practices in response to mishaps. Although this process can be formalized and carried out by experts, which is the apparent hope of the USG in enacting the current pause of research on dangerous respiratory pathogens [28], the uncertainty over the numbers is unlikely to quell the debate and could lead to further acrimony. We therefore believe that the value in risk-benefit calculations is not necessarily in a quantitative outcome per se but in the fact that the process will promote analysis and discussion of the variables involved, which in turn could improve experimental design and safety protocols. Given the degree of dissatisfaction with the status quo and the complexity of the issues, we believe a new approach to the problem is needed.

Having been involved in this area of science policy since the early days, we have given considerable thought to the issues. We believe that everyone can agree on some basic facts. First, studying infectious agents and toxins is important since this work can lead to new therapeutics, vaccines, and diagnostics. Second, the vast majority of scientists are law-abiding, ethical individuals who are unlikely to cause harm deliberately. Third, there are those who will engage in bioterror, as proved by the anthrax mailings, the poisoning of salad bars by the Rajaneeshee cult, and the attempted release of anthrax by the Aum Shinrikyo. Despite a reasonably precise definition, what we can’t seem to agree on is what constitutes DURC, mainly because the assessment involves judgment calls on risks and benefits [25].

We submit that the problem lies with the term “dual use” itself. Many useful everyday objects can be misused. An automobile can be loaded with explosives and turned into a bomb, and a kitchen knife can be used for murder. Consequently, some subtlety is required to evaluate what constitutes “dual use research of concern.” Scientists struggle with the idea that their work, which is aimed at benefitting mankind, might be misused. Finally, as noted above, the debate has changed in focus from biosecurity to biosafety, making DURC too narrow a concept going forward. We propose that it is time to redefine the problem and develop more acceptable terminology.

The question becomes, what is it we are truly worried about? First, there are individuals and groups who would like to engage in bioterrorism. While we cannot rule out that nation states might also be tempted to do so, we are hopeful that they abide by the Biological Weapons Convention. Second, there is the chance that, despite our best efforts, dangerous agents might accidentally be released. Attempts have been made to estimate the probability of an accident [24], and while there is disagreement about the actual numbers, the chances are clearly greater than zero. We are faced with risks of bioterror and biosafety breaches that we can only guess at or estimate. Similarly, we are faced with an inability to achieve a consensus about the likelihood of benefit, particularly because the benefits of any given experiment may not become evident until well into the future. We acknowledge that some risk is unavoidable and that progress often involves taking risks [29].

To us, the essential questions are, what experiments does humanity truly need and which risks are we willing to accept? The answers are not black or white, but what is required to answer these questions is more concrete. We believe that those scientists who are most closely associated with these experiments cannot make these determinations alone. A larger group of stakeholders must contribute, including the broader scientific community, public health experts, and ethicists, to name a few. We realize that engaging in such discussions could slow down progress or even discourage scientists from engaging in certain fields of research. However, if the decision is made that the work is not dangerous, might the good will that is garnered by obtaining the buy-in of a broad cohort be worth some delay, at least in the shorter term? In addition, as we learn more and gain experience discussing examples, the process could accelerate, similar to how experience with recombinant DNA has been used to facilitate its oversight.

We propose that rather than creating a new catchy term, we first acknowledge that there are risks, albeit ones that are difficult to quantify. Continued improvements in public health preparedness are necessary should an agent escape a lab either accidentally or deliberately. We note that such preparation serves a true “dual use” of allowing enhanced responses to naturally occurring outbreaks. Second, we acknowledge that while the vast majority of, if not all, life sciences research is beneficial, not all experiments have the same value to humankind. Reasonable individuals can disagree about what the value is, and there must be an attempt to reach a consensus about the level of benefit. We are convinced that ongoing discussion of these issues in and of itself may be the best protection against potential harm by ensuring that all the factors are amply considered and by demonstrating to the public that the scientific community is acting responsibly.

In summary, we face a crisis in the biological sciences comparable to the concerns about recombinant DNA prior to the Asilomar conference. We suggest a new synthesis based on five pillars that builds on the progress that has been made during the past decade:

1. Definition of the medical and scientific problems that need to be solved to protect humanity from pandemic threats. This would include efforts to understand the relationship between virulence and transmissibility, the parameters that allow zoonotic pathogens to jump to humans, and/or new approaches that would confer broad immune protection. In our view, stating such problems clearly could help catalyze solutions.
2. Acknowledgment that research has inherent risks that can be minimized but never fully abolished. Potential risks should be discussed and considered in the context of each specific experiment with the goal of fostering a culture of enhanced safety.
3. Acknowledgment that, although risks and benefits posed by certain experiments are difficult to quantify, efforts must be made to assess the risks and benefits to ensure that they are on the table and considered carefully, employing available tools that include accepted methods of risk-benefit analysis to optimize benefit and minimize risk.
4. Development of new biosafety approaches, including safer laboratory strains, careful attention to protocol, constant improvement of infrastructure, and vaccines to protect laboratory personnel when possible. With influenza, for example, enhanced efforts to develop a pan-strain vaccine could be a priority.
5. Creation of a national board to vet issues related to research with dangerous pathogens [25]. One possibility is to model a board after the Recombinant DNA Advisory Committee. Such a board should have microbiological, infectious disease, biosafety, and ethical expertise, which, combined with access to national security information, would allow better assessments of biosafety and biosecurity issues. As time passes, our hope would be that improvements in knowledge, the ability to combat or prevent diseases, and experience with containment will result in fewer and fewer projects being tagged as potentially dangerous. It is important to acknowledge that biosafety and biosecurity concerns transcend national interests, and perhaps the creation of such an entity in the US will galvanize the international community to follow suit.

We are cognizant that each of these pillars will require considerable discussion and debate, but together they provide a new framework for evaluating research, a framework that could ensure a safe and vigorous infectious diseases research enterprise.

## Abbreviations

CDC: Centers for Disease Control and Prevention
DURC: dual use research of concern
GOF: gain of function
IL-4: interleukin 4
NRC: National Research Council
NSABB: National Science Advisory Board for Biosecurity
SATL: Select Agents and Toxins List
USG: US government
